# Lower extremity necrotizing fasciitis with iliopsoas abscess secondary to perforated colon cancer: a diagnosis not to miss

**DOI:** 10.1093/jscr/rjad685

**Published:** 2023-12-30

**Authors:** Elias E Lahham, Mohammad I Alsahouri, Abdalrazeq A Ghweir, Qusai A Alsalah, Mohammad AlQadi, Nader Sarhan

**Affiliations:** Radiation Oncology Department, Augusta Victoria Hospital, Jerusalem 9511208, Palestine; Faculty of Medicine, Palestine Polytechnic University, Hebron 150, Palestine; Faculty of Medicine, Palestine Polytechnic University, Hebron 150, Palestine; Faculty of Medicine, Palestine Polytechnic University, Hebron 150, Palestine; Faculty of Medicine, Palestine Polytechnic University, Hebron 150, Palestine; General Surgery Department, Beit-Jala Hospital, Bethlehem 4322, Palestine; Faculty of Medicine, Palestine Polytechnic University, Hebron 150, Palestine; Shaare Zedek Medical Center, Jerusalem 9103102, Israel

**Keywords:** colon cancer, iliopsoas abscess, lower extremity, necrotizing fasciitis

## Abstract

Necrotizing fasciitis (NF) is a life-threatening soft tissue infection, typically caused by preexisting conditions such as trauma, complicated intraabdominal infections, or even small wounds. However, it is very rare for NF to occur as a result of perforated colon cancer (CC). Diagnosis primarily relies on clinical findings, imaging, and laboratory tests. Early diagnosis and treatment are crucial for patient survival. In this study, we present a case of an 82-year-old female a known case of CC diagnosed 1 month ago. She presented with hip pain persisting for 10 days duration, along with skin changes over the proximal anterolateral aspect of the thigh. The patient was diagnosed with NF associated with an iliopsoas abscess caused by perforated CC that was managed with surgical debridement, left hemicolectomy, and end colostomy along with broad-spectrum antibiotics.

## Introduction

Necrotizing fasciitis (NF) is an aggressive, rapidly progressive, life-threatening infection [1]. Dr B. Wilson coined this term for the first time in 1952 [2]. It can lead to necrosis in the skin, subcutaneous fat, and superficial and deep muscle fascia [3], and has an incidence of 0.5–15 cases in every 100 000 population [1]. Most patients with NF have preexisting medical conditions such as diabetes mellitus, immune suppression, liver and kidney diseases, and malignancy [4]. A previous study showed that only 16% of NF cases are secondary to perforated gastrointestinal tract malignancy [5]. Diagnosis primarily relies on clinical assessment [1]. Laboratory and imaging studies can aid in the diagnosis [6]. The mainstay treatment is early debridement, along with broad-spectrum antibiotics. In this report, we describe a case of NF secondary to perforated colon cancer (CC) in an 82-year-old female patient, who presented to our Emergency Department complaining of hip pain for 10 days. She was diagnosed based on clinical findings, imaging, and laboratory tests. The patient was managed with surgical intervention and intravenous broad-spectrum antibiotics. This report aims to alert surgeons to the importance of early diagnosis and treatment of NF and to consider CC perforation as one of the possible causes of NF, despite its rarity.

## Case presentation

**Figure 1:**
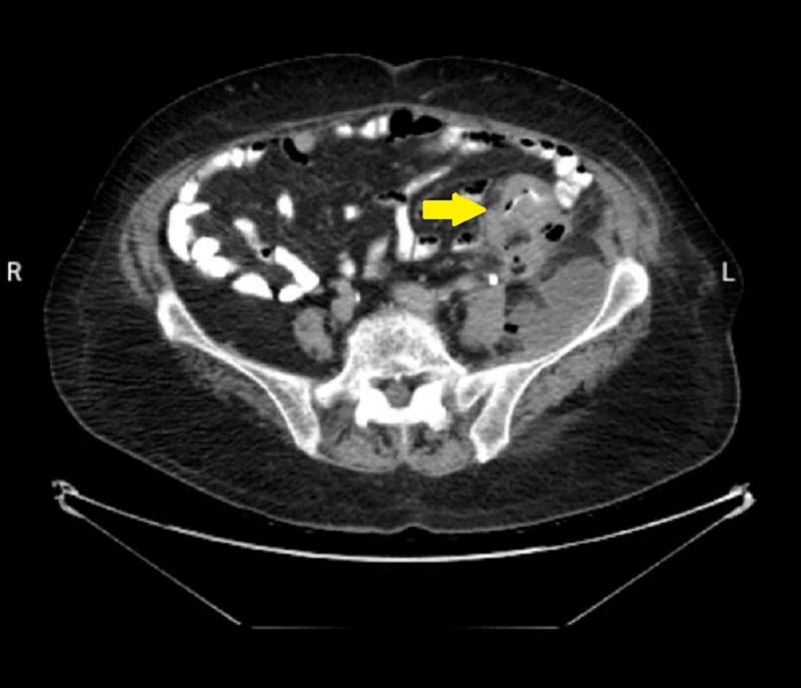
Abdominal CT scan with contrast showed the neoplasm and the presence of fluid collection (done at the time of the diagnosis of CC).

**Figure 2:**
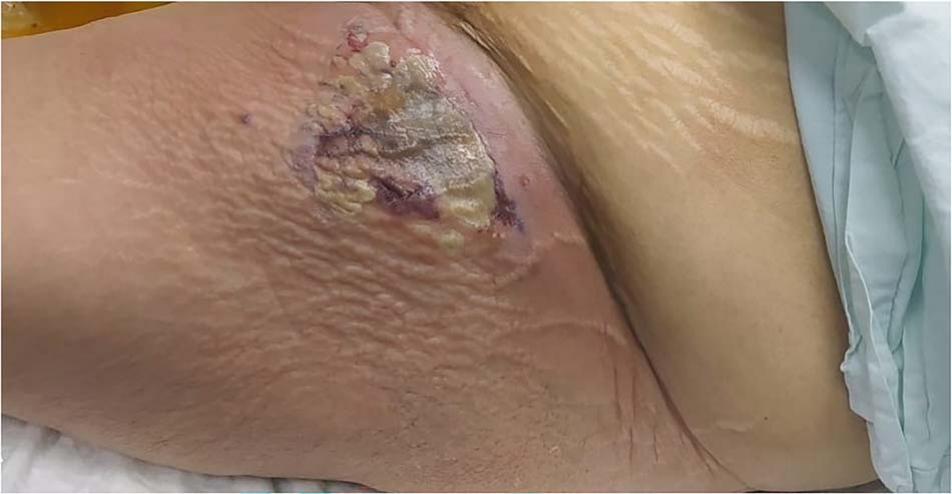
A large, tender erythematous swelling on the proximal anterolateral aspect of the left thigh. The swelling was fluctuant, with a black necrotic center.

An 82-year-old female patient with a known history of sigmoid colon adenocarcinoma diagnosed 1 month ago (Fig. 1), presented to the Emergency Department due to hip pain for 10 days, associated with abdominal pain, bloody diarrhea, and loss of appetite. The patient has a medical history of hypertension, and congestive heart failure with ejection fraction 20%. Her vitals were a temperature of 38.3°C, heart rate of 102/min, respiratory rate of 22/min, blood pressure of 90/60 mm Hg, and pulse oximetry of 94%. On examination, there was a large, tender erythematous swelling on the proximal anterolateral aspect of the left thigh. The swelling was fluctuant, with a black necrotic center (Fig. 2). Laboratory tests revealed an elevated white blood cell count, C-reactive protein level, and signs of acute kidney injury (Table 1).

**Table 1:** Laboratory values on admission.

Laboratory test	Laboratory values on admission	Normal values
Hemoglobin (HB)	9.2 g/dl	(12–16 g/dl)
Creatinine (Cr)	1.9 mg/dl	(0.5–0.9 mg/dl)
Potassium (K)	4.04 mmol/L	(3.5–5.2 mmol/L)
Sodium (Na)	135 mmol/L	(135–145 mmol/L)
White blood cell (WBC)	33.8 cells/L	(4.6–11 cells/L)
C-reactive protein (CRP)	292 g/dl	(0–5 g/dl)
Blood urea nitrogen (BUN)	51.3 mg/dl	(8–23 mg/dl)
Random blood sugar (RBS)	152 mg/dl	(74–110 mg/dl)

**Figure 3:**
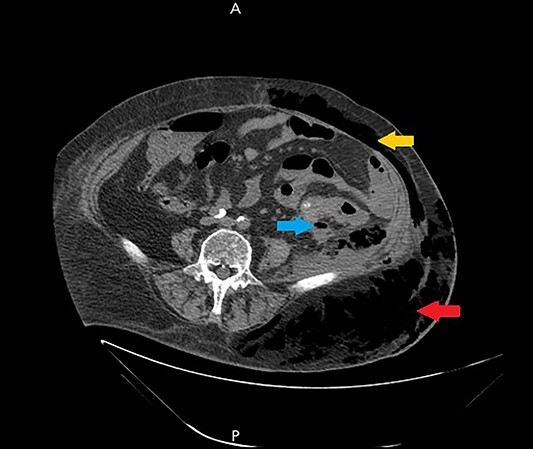
A non-contrast abdomen and pelvis CT scan, which shows a large amount of subcutaneous emphysema within the left flank soft tissues (lower arrow) that wrapped anteriorly along the left lower lateral abdominal wall into the anterior subcutaneous soft tissues of the left lower quadrant (upper arrow). There was also a small tract of air extending from the left retro peritoneum posterior to the left kidney and extending along the left iliopsoas muscle inferiorly (middle arrow).

**Figure 4:**
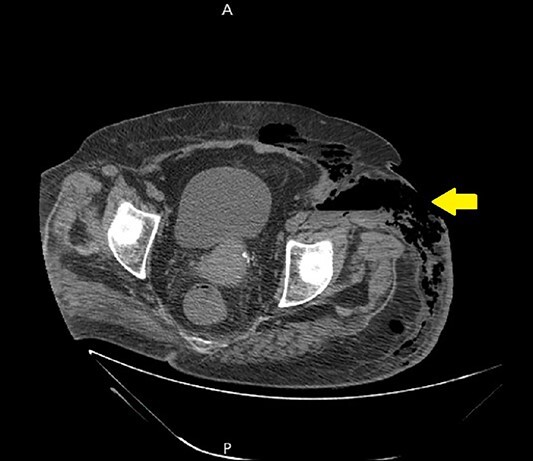
A non-contrast abdomen and pelvis CT scan, which shows a skin defect with underlying abscess collection and air–fluid level, with significant subcutaneous air collection.

**Figure 5:**
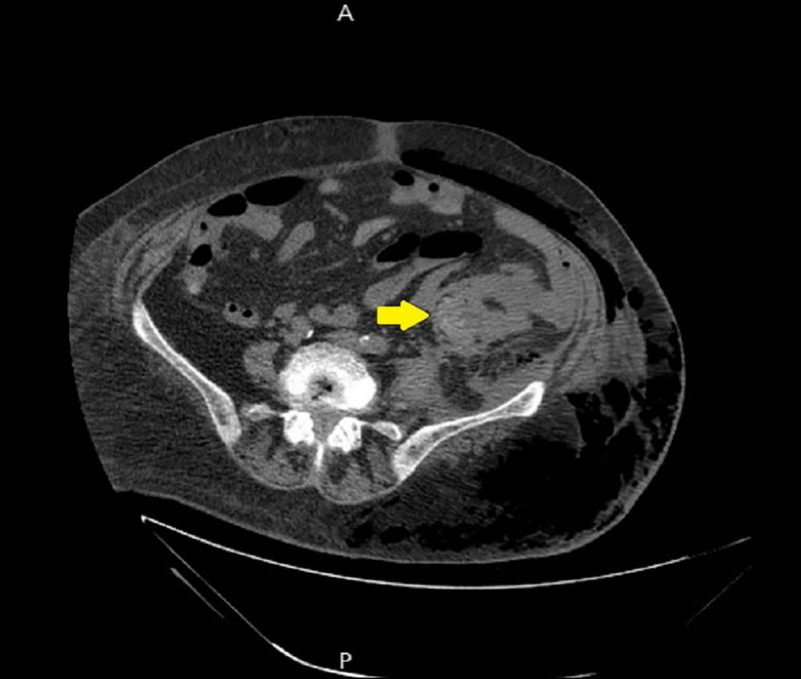
A non-contrast abdomen and pelvis CT scan, which shows a soft tissue mass lesion at the distal descending colon.

Ultrasound showed extensive subcutaneous soft tissue edema with hyperechoic areas seen in the proximal anterolateral part of the thigh. The initial diagnosis of the condition was a left iliopsoas abscess. Due to the severity of the patient’s symptoms, a non-contrast abdomen and pelvis computerized tomography (CT) scan was done (Fig. 3), which showed a large amount of subcutaneous emphysema within the left flank soft tissues that wrapped anteriorly along the left lower lateral abdominal wall with a small tract of air extending from the left retroperitoneum posterior to the left kidney and extending along the left iliopsoas muscle inferiorly. A skin defect with the underlying abscess collection and air–fluid level was noted (Fig. 4). Another CT scan section saw a soft tissue mass lesion at the distal descending colon (Fig. 5). The abscess drainage was done under CT guidance.

With a Laboratory Risk Indicator for Necrotizing Fasciitis (LRINEC) score of 10 and examination findings, a diagnosis of NF of the left thigh was made. Then, the patient was transferred immediately to the operating room. Extensive debridement of the fascia, muscle, soft tissue, and skin was done. An exploratory laparotomy was done due to the suspicion of perforated CC. Intraoperatively, after an extensive debridement, a distal descending colon perforation was found, which was tightly adhered to the surrounding tissues and left retroperitoneum. A fistula was identified, which was the cause of the iliopsoas abscess. A left hemicolectomy with a colostomy was done. Following surgery, the patient underwent critical care for septic shock and the open drainage site lavage. Despite antibiotic treatment and aggressive resuscitation, the patient died from septic shock 4 days later.

## Discussion

NF is a severe form of skin and soft tissue infection characterized by necrosis of the fascia, muscles, and subcutaneous tissue [5]. It often follows trauma, complicated intraabdominal infections, or small wounds [7]. NF can be categorized based on microbiological subtypes: Type I involves a polymicrobial infection, indicating the presence of both anaerobic and aerobic bacterial species, Type II is characterized by a monomicrobial infection, with Group A Streptococcus and Clostridium being the most frequent causative agents [8]. In cases of NF, the typical clinical presentation includes a patient’s reported history of pain, swelling, and fever, along with noticeable tenderness and redness at the affected site during a physical examination [1, 6]. NF on the lower extremities or abdominal wall without an obvious cutaneous source, an intra-abdominal cause should be considered [9]; surprisingly, only 16% of NF cases are caused by a perforated gastrointestinal tract secondary to malignancy [5]. The prevalence of perforated CC is between 3% and 10% [10], and the development of an intra-abdominal abscess is exceedingly rare, occurring in only 0.3%–0.4% of cases [11]. A psoas abscess from CC is typically preceded by cancer perforation and fistula development [12]. Colon perforation leading to NF of the thigh has been explained by the entry of fecal matter through the femoral sheath, psoas sheath, femoral canal, obturator foramen, or sciatic notch [9, 13]. Diagnosis primarily relies on clinical assessment [1]. The preferred imaging technique for diagnosing NF is a CT scan, which typically reveals edema and fluid along the fascial planes. In some cases, it is also possible to see gas tracking along the fascial planes [1]. The LRINEC score is a reliable tool that can aid in the clinical diagnosis of NF [14]. Management of NF requires broad-spectrum antibiotics, emergent operative debridement, and intensive care for sepsis [1, 15]. Without debridement, the mortality rate is nearly 100% [6], and a delay in surgical debridement is associated with a mortality rate of up to 70% [7]. Even with appropriate care, the death rate in NF can reach 25% [15].

## Conclusion

Perforation of CC is a rare cause of NF. In our case, the patient presented with hip pain due to an iliopsoas abscess that resulted from perforated CC and fistula formation. Subsequently, the iliopsoas abscess led to NF. Despite surgical debridement, drainage, and antibiotic treatment, the patient succumbed to sepsis. Our study aims to raise awareness among surgeons regarding the risk of CC perforation causing iliopsoas abscess and NF. Early detection and intervention can potentially reduce the mortality rate associated with NF.

## Data Availability

The data used to support the findings of this study are included in the article.
